# Determining the Optimal Parameters for Scoring Systems to Predict Postoperative Bleeding After Diabetic Coronary Artery Bypass Surgery

**DOI:** 10.21470/1678-9741-2022-0178

**Published:** 2026-01-29

**Authors:** Engin Akgul, Abdulkerim Ozhan

**Affiliations:** 1 Department of Cardiovascular Surgery, Kütahya Health Science University Evliya Çelebi Training and Research Hospital, Kutahya, Turkey

**Keywords:** Creatinine, Platelet Count, Hematocrit, Body Mass Index, Glomerular Filtration Rate, Postoperative Hemorrhage, Drainage, Coronary Artery Bypass

## Abstract

**Introduction:**

Postoperative bleeding increases morbidity and mortality. We aimed to review the scoring systems used to predict massive bleeding after isolated coronary artery bypass grafting in diabetic patients and determine the parameters of the new scoring system - the Optimum Risk Score for Bleeding (ORS).

**Methods:**

Two hundred ninety-seven diabetic patients who underwent isolated coronary artery bypass operation between 2017 and 2019 were reviewed. The patients were grouped according to amount of drainage (> 850 mL/day) and the European Multicenter Study on Coronary Artery Bypass Grafting (E-CABG) bleeding severity grade. Previously identified risk factors and scoring systems (Papworth, WILL-BLEED, Association of Cardiothoracic Anesthetists perioperative risk of blood transfusion [ACTA-PORT], Transfusion Risk and Clinical Knowledge [TRACK], and Transfusion Risk Understanding Scoring Tool [TRUST]) were analyzed.

**Results:**

Papworth was better predictive for E-CABG bleeding grades 2 - 3. WILL-BLEED, ACTA-PORT, TRACK, and TRUST had no discriminatory value in terms of E-CABG bleeding grades 2 - 3. Among the parameters in the scoring systems, gender, preoperative hemoglobin (or hematocrit) value, preoperative platelet count, use of antiplatelets until less than five days prior to the operation, and preoperative creatinine (or estimated glomerular filtration rate) values should be included in the scoring system we aim to establish in the future, the ORS.

**Conclusion:**

The current scoring systems do not provide satisfactory results in predicting postoperative bleeding. Female gender, lower body mass index, and preoperative platelet count were associated with increased postoperative bleeding. There is a need for an ORS which gives more precise results in predicting postoperative bleeding.

## INTRODUCTION

**Table:** 

Abbreviations, Acronyms & Symbols				
ACT	= Activated clotting time		HT	= Hypertension
ACTA-PORT	= Association of Cardiothoracic Anesthetists perioperative risk of blood transfusion		IABP ICU	= Intra-aortic balloon pump = Intensive care unit
Adj.	= Adjusted		IQR	= Interquartile range
ASA	= Acetylsalicylic acid		LE	= Lower end
BMI	= Body mass index		LMWH	= Low molecular weight heparin
BSA	= Body surface area		LR	= Likelihood ratio
CABG	= Coronary artery bypass grafting		MPV	= Mean platelet volume
CI	= Confidence interval		OAD	= Oral antidiabetic drug
CPB	= Cardiopulmonary bypass		OR	= Odds ratio
CPD	= Chronic pulmonary disease		ORS	= Optimum Risk Score for Bleeding
DM	= Diabetes mellitus		Plt.	= Platelet
E-CABG	= European Multicenter Study on Coronary Artery Bypass Grafting		RBC ROC	= Red blood cell = Receiver operating characteristic
eGFR	= Estimated glomerular filtration rate		SD	= Standard deviation
EuroSCORE	= European System for Cardiac Operative Risk Evaluation		SE	= Standard error
FFP	= Fresh frozen plasma		TRACK	= Transfusion Risk and Clinical Knowledge
Fpx	= Fondaparinux		TRUST	= Transfusion Risk Understanding Scoring Tool
Hb	= Hemoglobin		UE	= Upper end
HRs	= Hazard ratios		UFH	= Unfractionated heparin

In the light of the studies we have found in the literature, we now know that postoperative bleeding is a serious cause of mortality and morbidity that disrupts the function and structural integrity of organs^[1,2]^. Bleeding can occur due to many different surgical procedures. However, the fact that atherosclerotic patients receive antiplatelet treatment is significant for cardiac surgeons, whose working area consists of blood and the circulatory system^[3]^. Conditions such as hemodilution, number and structural changes in platelets, and hypothermia, which occur during cardiac operations performed with a heart-lung machine, may cause impairment of the coagulation system^[4]^. Female gender and lower body mass index (BMI) were also found to be associated in postoperative bleeding after cardiac surgery^[5-7]^. The need for blood transfusion in cardiac operations varies between 20% and 80%^[5]^. This wide transfusion margin is because postoperative hemorrhagic drainage is considered normal for a certain period and amount unless the patient is hemodynamically unstable^[1,3]^. While drains that do not disrupt hemodynamics do not require re-exploration, bleeding that causes hypovolemia can lead to permanent damage to vital organs and life-threatening consequences^[8]^. As it requires more fluid replacement, postoperative volume loss increases the need for transfusion and related complication rates. In addition, postoperative bleeding has also been shown to increase intensive care unit (ICU) stay, infection rate, intubation time, and hospital costs^[9,10]^.

Postoperative bleeding may occur due to coagulopathy or surgical technique and related problems. Whatever the reason, guidelines on blood management have been established^[11]^ based on numerous studies performed on coronary bypass operations over the years. For successful postoperative bleeding control, the process needs to begin from the preoperative period^[5]^. A thorough analysis of preoperative demographic data and drug use will help the postoperative process to proceed more smoothly. Detection of anemia, initiation of erythropoietin therapy, and practices to increase preoperative blood reserve, such as blood donation, may help reduce the need for postoperative transfusion^[5]^. The need for transfusion can be significantly reduced with perioperative cell salvage methods^[12]^.

Less postoperative drainage results in the use of less blood product transfusions. Thus, complications related to blood product transfusion are also reduced. It is for this reason that scoring systems have been defined for bleeding modality. The ones we investigate in this study are the Papworth, which was developed by Vuylsteke et al.^[13]^, WILL-BLEED, by Biancari et al.^[3]^, Association of Cardiothoracic Anesthetists perioperative risk of blood transfusion (ACTA-PORT), by Klein et al.^[6]^, Transfusion Risk and Clinical Knowledge (TRACK), by Ranucci et al.^[14]^, and Transfusion Risk Understanding Scoring Tool (TRUST) by Alghamdi et al.^[15]^.

The reason we chose this patient population is because diabetes causes microvascular endothelial dysfunction^[16,17]^, and impairments have been shown in the fibrinolytic system and coagulation factor functions in diabetic patients^[18]^. Endothelial damage, increased oxidative stress, chronic inflammation, and impaired fibrinolytic system seen in patients with DM are its main causes^[18]^.

Our aim in this study was to review the scoring systems that can be used to predict early massive bleeding after coronary artery bypass grafting (CABG) in diabetic patients undergoing isolated coronary bypass surgery and determine the parameters of the Optimum Risk Score for Bleeding (ORS), which is currently our ongoing project. Our aim in this project is to objectively determine the effective parameters in postoperative bleeding and to put ORS into clinical use.

## METHODS

We organized our study as a retrospective archive scan. Following the permission we received from the local ethics committee to scan the patient files (decision nº: 2020/85), patients who underwent isolated coronary bypass operation at Kutahya Health Sciences University Evliya Celebi Training and Research Hospital between 2017 and 2019 were examined. Inclusion criteria comprised patients who were diagnosed with diabetes mellitus and underwent an isolated coronary bypass operation for the first time. Patients who underwent beating heart surgery, presented to the emergency department with acute myocardial infarction and started ticagrelor treatment, and those 11 patients who died within the first 24 postoperative hours were excluded. When the files of patients who died within the first 24 hours were examined, we saw that only three of these 11 patients underwent re-exploration, but no information about bleeding was given in the notes of the re-exploration surgery. Malignant arrhythmia and/or diastolic heart failure were shown as the cause of mortality. Since we could not obtain clear information about the cause of mortality, these patients were excluded from the study. Death within the first 24 hours is a competing risk factor. Surgical technique-induced bleeding, such as bleeding due to surgical error and non-ligatured vascular structures, were found in the operation notes, and these patients were excluded from our study. In addition, the amount of drainage before re-exploration was used for the scoring systems in patients who were explored due to bleeding. Demographic characteristics and hematological parameters were determined according to the blood samples obtained preoperatively, at the closest date to the operation day. Since our study is focused on early postoperative bleeding, it includes the first 24 hours of postoperative follow-up, because bleeding and related complications are likely to occur in the early period. The amount of drainage and blood products used in the first 24 postoperative hours were recorded. Since this was a retrospective study, the need to obtain informed consent was exempted.

This study was based on a prospective multicenter study in Europe, the European Multicenter Study on Coronary Artery Bypass Grafting (E-CABG) (clinicaltrials.gov identifier: NCT02319083). The bleeding scores (Papworth, WILL-BLEED, TRACK, and TRUST) were compared with the E-CABG bleeding grades and analyzed to find which was more convenient for our patient population. In addition, significant parameters were determined, and it was aimed to lay the foundations of the ORS by using these parameters in larger studies.

E-CABG is a multicenter study conducted across six European countries (Germany, Italy, England, France, Sweden, and Finland) and 16 centers^[19]^. In this study, risk was calculated by scoring after grouping according to the blood products and amounts transfused.

### Clinical Management

In our center, cardiac operations were performed under general anesthesia (fentanyl 35 µg/kg, pancuronium 0.1 mg/kg) with positive pressure ventilation. Median sternotomy was performed in all patients, and aorto-right atrial cannulation method was preferred. Before switching to cardiopulmonary bypass (CPB), 3 mg/kg heparin was administered to the patients, and additional heparin dose was given, if necessary, to keep activated clotting time (ACT) > 480 s. When switching to CPB, antegrade or antegrade + retrograde crystalloid solutions were used as prime solutions according to the patient’s BMI. Systemic hypothermia was achieved by cooling the patient to 32°C. During the surgical procedure, blood accumulated in the thoracic cavity and pericardial area was collected in the reservoir and reinfused to the patient. However, unfortunately, methods such as cell saver could not be used. The cardiac operation was completed, and bleeding was controlled with protamine sulphate (3.1 mg/kg) administration to keep ACT < 120 s during weaning from CPB.

The left hemithorax was opened with left pleural incision in all patients. The right hemithorax may have been opened in some patients, however, bleeding was monitored in the ICU with 32 French drains placed in all hemithoracic cavities and 36 French drains placed in the mediastinum. After the sternum was closed with sternal wires, the subcutaneous and skin tissues were closed, and the intubated patient was transported to the ICU. In our center, the surgical team is responsible for intensive care of the patients. The patients were extubated in the ICU based on the extubation criteria. According to the institution’s protocol, patients with high volumes of drainage and/or hemodynamic instability were not extubated. In cases where the drainage was voluminous enough to impair hemodynamics, the patients were re-explored for bleeding revision. The use of fresh frozen plasma (FFP) was considered if international normalized ratio was > 1.7 or excessive bleeding was observed while the chest was open or if there was 2 mL/kg.h drainage after chest closure. If the central venous pressure was < 8 mmHg or when the patient had more drainage than expected, colloid solutions can be used instead of FFP, but FFP is primarily used due to clinical preference. Erythrocyte transfusion was performed when hemoglobin (Hb) < 8 gm%. Platelet suspensions were administered according to the platelet count in the hemogram obtained postoperatively as the patient entered the ICU.

Descriptive parameters of the scoring systems used in the study are shown in Table 1.

**Table 1:** Descriptive parameters of the scoring systems used in the study.

TRUST Score	Score	TRACK Score	Score
Hb < 13.5 g/dl	1	Age > 67 years	6
Weight < 77 kg	1	Weight: < 60 kg (female)	2
		< 85 kg (male)	
Female sex	1	Female sex	4
Age > 65 years	1	Complex surgery	7
Nonelective surgery	1	Hematocrit (continuous)	1 point per each value (%) below 40%
Creatinine > 1.36 mg/dl	1		
Previous cardiac surgery	1		
Non isolated operation	1		
WILL-BLEED Score	Score	Papworth Score	Score
LMWH, UFH, Fpx usage	1	Surgery priority: urgency/emergency	1
Potent antiplatelet drug pause	2	Surgery type: other than CABG or single valve	1
Female sex	2	Aortic valve disease: stenosis, regurgitation, or both	1
Acute coronary syndrome	2	BMI: < 25	1
Anemia (female < 120 g/L, male < 130 g/L)	3	Age ≥ 75 years	1
eGFR < 45 mL/min/173 m^2^	3		
Critical preoperative state	5		
E-CABG bleeding classification	Intervention for treatment of bleeding		Additive Score
Grade 0	No transfusion of blood products except 1 unit of RBCs		0
Grade 1	Transfusion of platelets		2
	Transfusion of fresh frozen plasma or Octaplas™		3
	Transfusion of 2 - 4 units of RBCs		3
Grade 2	Transfusion of 5 - 10 units of RBCs		5
	Reoperation for bleeding		5
Grade 3	Transfusion of > 10 units of RBCs		7

BMI=body mass index; E-CABG=European Multicenter Study on Coronary Artery Bypass Grafting; eGFR=estimated glomerular filtration rate; Fpx=fondaparinux; Hb=hemoglobin; LMWH=low molecular weight heparin; RBCs=red blood cells; TRACK=Transfusion Risk and Clinical Knowledge; TRUST=Transfusion Risk Understanding Scoring Tool; UFH=unfractionated heparin

### Statistical Analysis

Statistical analyses were performed using Jamovi Statistics (version 1.2.27 solid) software. Shapiro-Wilk test, histograms, and Q/Q plots were used to identify the distribution patterns. Nominal variables were presented as number and percentage, normally distributed continuous variables were presented as mean and standard deviation, and non-normally distributed continuous variables were presented as median and (Q1,Q3). In addition to descriptive statistics, Chi-square test was used for nominal values in the comparison of groups, independent samples *t*-test was used for comparison of parametric data, and Mann-Whitney U test was used for comparison of nonparametric data. Significant factors in univariate analysis were carried onto multivariate logistic regression analysis and reported as hazard ratios (HRs) and adjusted (adj.) HRs. To create easily calculable risk scores, continuous variables were transformed into categorical ones according to optimal cutoff values found out by receiver operating characteristic (ROC) curve analysis. Then, simple scores were created with these significant variables. *P*-value < 0.05 was considered statistically significant.

## RESULTS

This study included the data of 297 diabetic patients who underwent isolated coronary bypass surgery. Since our study focuses entirely on postoperative bleeding, patients who died due to any other reason within the first 24 postoperative hours were excluded from the study, along with patients who were re-explored for any reason other than bleeding. We based our study on the E-CABG study and evaluated the bleeding prediction scores accordingly. In addition, we examined the average drainage amount of the patients and analyzed the data.

The patients were divided into two groups according to their E-CABG grades (Table 2). E-CABG grades 0 and 1 (n = 260) were evaluated in the same group, just as grades 2 and 3 (n = 37). Grades 2 - 3 patients had lower BMI (*P* < 0.001), higher drainage amounts in the first 24 postoperative hours (*P* < 0.001), higher postoperative creatinine values (*P* = 0.02), higher amounts of postoperative blood product transfusion (red blood cell [RBC] transfusion [*P* < 0.001], FFP transfusion [*P* < 0.001], and platelet transfusion [*P* < 0.001]), and a higher ratio of female patients (*P* = 0.006).

**Table 2:** Determination of demographic characteristics and hematological parameters according to E-CABG.

Variables	E-CABG Grade				*P*-value
	Grades 0 - 1 (n = 260)		Grades 2 - 3 (n = 37)		
	Mean ± SD	Median (IQR)	Mean ± SD	Median (IQR)	
Age (years)	63.4 ± 9.3	64.5 (58-69)	64.2 ± 9.3	65.0 (57.0-69.0)	0.647
Postoperative drainage (0 - 24 h)	600 ± 218	550 (450-750)	1332 ± 453	1450 (1100-1600)	< .001
Preoperative hemoglobin (g/dl)	13.4 ± 1.9	13.6 (12.0-15.0)	13.9 ± 1.7	14.2 (13.0-14.9)	0.204
Preoperative hematocrit (%)	39.1 ± 5.7	39.4 (35.0-43.8)	40.3 ± 5.0	41.0 (38.2-43.5)	0.196
Preoperative platelet (10^3/ul)	248 ± 77.4	237 (198-290)	229 ± 102	222 (190-252)	0.044
Preoperative MPV (fl)	9.63 ± 1.05	9.60 (8.9-10.3)	9.81 ± 1.18	9.90 (8.9-10.4)	0.329
Preoperative creatinine (mg/dl)	1.09 ± 0.51	1.00 (0.86 - 1.16)	1.1 ± 0.33	1.04 (0.90 - 1.22)	0.290
Preoperative creatinine clearance	80.9 ± 27.0	79.3(64.8 - 98.3)	73.8 ± 22.8	71.7 (57.1 - 88.2)	0.137
Postoperative creatinine (mg/dl)	1.15 ± 0.51	1.06 (0.93 - 1.22)	1.35 ± 0.34	1.31 (1.20 - 1.43)	0.020
eGFR (ml/dk/1.73m^2^)	64.5 ± 20.8	63.3 (50.2 - 76.4)	58.6 ± 19.7	55.3 (44.4 - 71.6)	0.104
BMI	28.1 ± 4.0	28.0 (25.6 - 30.5)	25.3 ± 3.3	25.5 (23.5 - 26.4)	< 0.001
BSA	1.85 ± 0.16	1.85 (1.74 - 1.96)	1.83 ± 0.13	1.86 (1.72 - 1.92)	0.396
EuroSCORE II	3.92 ± 5.78	2.08 (1.34 - 3.84)	4.06 ± 5.03	2.35 (1.48 - 4.03)	0.316
Ejection fraction	48.8 ± 10.0	50 (40 - 60)	48.4 ± 10.2	50 (40 - 55)	0.828
RBC transfusion	1.4 ± 1.0	1 (1 - 2)	4.6 ± 1,8	5 (1 - 2)	< .001
FFP transfusion	2.7 ± 1.2	3 (2 - 3)	4.8 ± 1.6	5 (2 - 3)	< .001
Platelet transfusion	0.2 ± 0.4	0 (0 - 0)	0.9 ± 1.0	1 (0 - 1)	< .001
Variables	E-CABG Grade				*P*-value
	Grades 0 - 1 (n = 260)		Grades 2 - 3 (n = 37)		
	n	%	n	%	
Gender (female)	193	74.2	35	94.6	0.006
Re-exploration for bleeding	0	0	29	78.4	< .001
DM (OAD)	127	48.8	17	45.9	0.741
DM (insulin dependent)	133	51.2	20	54.1	0.741
Hypertension	112	43.1	17	45.9	0.742
Peripheral vascular disease	60	23.1	8	21.6	0.844
Pulmonary HT (> 60 mmHg)	19	7.3	2	5.4	0.673
Preoperative IABP	18	6.9	4	10.8	0.398
CPD	42	16.2	2	5.4	0.085
Emergency surgery	23	8.8	4	10.8	0.697

BMI=body mass index; BSA=body surface area; CPD=chronic pulmonary disease; DM=diabetes mellitus; E-CABG=European Multicenter Study on Coronary Artery Bypass Grafting; eGFR=estimated glomerular filtration rate; EuroSCORE=European System for Cardiac Operative Risk Evaluation; FFP=fresh frozen plasma; HT=hypertension; IABP=intra-aortic balloon pump; IQR=interquartile range; MPV=mean platelet volume; OAD=oral antidiabetic drug; RBC=red blood cell; SD=standard deviation

Patients were grouped according to the amount of drainage from thoracic tubes and re-evaluated (Table 3). The median drainage was 600 ml (450 ml and 850 ml for 25% and 75% percentiles, respectively). A cutoff value of 850 ml (75% percentile) indicated massive drainage in our study group, and when grouped accordingly, preoperative platelet count (*P* < 0.001), creatinine clearance (*P* = 0.025), estimated glomerular filtration rate (eGFR) (*P* = 0.004), and BMI (*P* < 0.001) values were significantly higher among patients with drainages < 850 ml/day. Postoperative creatinine values (*P* = 0.008) and female gender (*P* = 0.001) were higher in patients with a drainage > 850 ml/day.

**Table 3:** Determination of demographic characteristics and hematological parameters according to drainage amount.

Variables	Groups				*P*-value
	Drainage < 850 ml/day (n = 218)		Drainage ≥ 850 ml/day (n = 79)		
	Mean ± SD	Median (IQR)	Mean ± SD	Median (IQR)	
Age (years)	63.2 ± 9.3	65 (57 - 69)	64.4 ± 9.1	64 (58 - 70)	0.343
Postoperative drainage (0 - 24 h)	525 ± 161	550 (400 - 650)	1149 ± 338	1000 (900 - 1400)	< .001
Preoperative hemoglobin (g/dl)	13.5 ± 1.88	13.7 (12.0 - 15.0)	13.5 ± 1.97	13.8 (12.3 - 14.9)	0.908
Preoperative hematocrit (%)	39.3 ± 5.6	39.5 (35.0 - 44.0)	39.1 ± 5.7	39.7 (36.0 - 43.4)	0.850
Preoperative platelet (10^3/ul)	253 ± 76	242 (206 - 296)	227 ± 91	215 (184 - 259)	0.001
Preoperative MPV (fl)	9.68 ± 1.02	9.65 (8.93 - 10.4)	9.58 ± 1.18	9.40 (8.8 - 10.1)	0.472
Preoperative creatinine (mg/dl)	1.06 ± 0.50	1.00 (0.84 - 1.16)	1.15 ± 0.48	1.05 (0.90 - 1.19)	0.052
Preoperative creatinine clearance	82.1 ± 26.9	81.9 (64.8 - 99.4)	74.3 ± 23.8	73.6 (61.6 - 91.5)	0.025
Postoperative creatinine (mg/dl)	1.13 ± 0.50	1.05 (0.91 - 1.22)	1.30 ± 0.49	1.20 (1.08 - 1.35)	0.008
eGFR (ml/dk/1.73m^2^)	65.9 ± 21.1	63.6 (50.9 - 78.5)	58.0 ± 18.8	56.8 (47.0 - 71.3)	0.004
BMI	28.4 ± 4.1	28.1 (25.7 - 30.9)	26.1 ± 3.1	25.9 (23.9 - 27.8)	< .001
BSA	1.85 ± 0.16	1.85 (1.73 - 1.96)	1.85 ± 0.13	1.85 (1.75 - 1.94)	0.889
EuroSCORE II	3.88 ± 5.15	2.09 (1.34 - 3.98)	4.09 ± 6.98	2.10 (1.44 - 3.64)	0.921
Ejection fraction	48.6 ± 10.0	50 (40 - 60)	48.9 ± 10.1	50 (40 - 60)	0.828
RBC transfusion	1.3 ± 1.0	1 (1 - 2)	3.1 ± 1.9	3 (2 - 5)	< .001
FFP transfusion	2.7 ± 1.2	2 (2 - 3)	3.9 ± 1.6	4 (3 - 5)	< .001
Platelet transfusion	0.2 ± 0.4	0 (0 - 0)	0.5 ± 0.8	0 (0 - 1)	< .001
Variables	Groups according to drainage				*P*-value
	Drainage < 850 ml/day (n = 218)		Drainage ≥ 850 ml/day (n = 79)		
	n	%	n	%	
Gender (female)	157	72.0	71	89.9	0.001
Re-exploration for bleeding	1	0.5	28	35.4	< .001
DM (OAD)	103	47.2	41	51.9	0.479
DM (insulin dependent)	115	52.8	38	48.1	0.479
Hypertension	96	44.0	33	41.8	0.728
Peripheral vascular disease	52	23.9	16	20.3	0.514
Pulmonary HT (> 60 mmHg)	17	7.8	4	5.1	0.417
Preoperative IABP	16	7.3	6	7.6	0.941
CPD	35	16.1	9	11.4	0.318
Emergency surgery	21	9.6	6	7.6	0.589

BMI=body mass index; BSA=body surface area; CPD=chronic pulmonary disease; DM=diabetes mellitus; eGFR=estimated glomerular filtration rate; EuroSCORE=European System for Cardiac Operative Risk Evaluation; FFP=fresh frozen plasma; HT=hypertension; IABP=intra-aortic balloon pump; IQR=interquartile range; MPV=mean platelet volume; OAD=oral antidiabetic drug; RBC=red blood cell; SD=standard deviation

Significant variables in univariate analysis or those confirmed as significant in clinical practice were carried onto multivariate analysis (Table 4). Risk factors for E-CABG grades 2 - 3 were analyzed in Models 1A and 2A, and risk factors for massive postoperative drainage were analyzed in Models 1B and 2B (Model 2A: Nagelkerke R^2^ 14.5%, accuracy 87.2%; Model 2B: Nagelkerke R^2^ 15.1%, accuracy 74.4%). Accordingly, female gender (*P* = 0.01) and BMI (*P* < 0.001) appeared as independent predictors of being in the E-CABG grades 2 - 3 group. According to the multivariate analysis, the bleeding probability is 4.8 (adj. HR: 4.85, 95% confidence interval [CI]: 1.11 - 22.2) times higher in females than males, and one unit increase in BMI decreases the likelihood of being in E-CABG grades 2 - 3 group by almost 20 percent (adj. HR: 0.81, 95% CI: 0.73 - 0.92). After finding the predictors, BMI was categorized according to the ROC analysis (cutoff value: 27 points) to form a simply usable score (Table 5). The rate of being in the E-CABG grades 2 - 3 group was 2.04% (1/49) in male patients with BMI > 27, so we considered these patients in the low-risk group. Moreover, this rate was 4.51% (6/133) in the patient group in which only one of them (female or BMI < 27) was present. In this group, which we considered medium risk, the probability of being in the E-CABG grades 2 - 3 group did not increase statistically significantly despite the increase in the ratio (HR: 2.27, 95% CI: 0.26 - 19.33, *P* = 0.4539). However, in the group of females with BMI < 27 (the high-risk group), this ratio was about 26.15% (30/115), indicating almost a 17-time increase in the likelihood of being in the E-CABG grades 2 - 3 group (HR: 16.9, 95% CI: 2.24 - 128.1, *P* = 0061).

**Table 4:** Determining the variables with multivariate analysis which were found statistically significant in univariate analysis or confirmed to be significant in clinical practice.

Variables	Multivariate analysis (E-CABG grades 2 - 3)			
	Model 1A		Model 2A	
	OR (95% CI)^*^	LR test statistics	OR (95% CI)	LR test statistics
Age (years)	1.02 (0.97 - 1.06)^NS^	X^2^_(1)_ = 0.62, *P* = 0.43	Removed	Removed
Gender (female)	4.04 (0.80 - 20.41)^NS^	X^2^_(1)_ = 3.58, *P* = 0.06	4.85 (1.11 - 22.2)^*^	X^2^_(1)_ = 6.53, *P* = 0.01
Preoperative Hb	1.11 (0.90 - 1.37)^NS^	X^2^_(1)_ = 0.98, *P* = 0.32	Removed	Removed
Preoperative Plt.	1.00 (0.99 - 1.00)^NS^	X^2^_(1)_ = 0.56, *P* = 0.45	Removed	Removed
eGFR	1.00 (0.98 - 1.02)^NS^	X^2^_(1)_ = 0.001, *P* = 0.97	Removed	Removed
BMI	0.81 (0.72 - 0.91)^***^	X^2^_(1)_ = 14.4, *P* < .001	0.81 (0.73 - 0.92)^***^	X^2^_(1)_ = 14.0, *P* < 0.001
Emergency surgery	1.59 (0.49 - 5.22)^NS^	X^2^_(1)_ = 0.55, *P* = 0.46	Removed	Removed
Variables	Multivariate analysis (drainage ≥ 850 ml/day)			
	Model 1B		Model 2B	
	OR (95% CI)^*^	LR test statistics	OR (95% CI)	LR test statistics
Age (years)	1.01 (0.98 - 1.04)^NS^	X^2^_(1)_ = 0.289, *P* = 0.59	Removed	Removed
Gender (female)	2.17 (0.88 - 5.30)^NS^	X^2^_(1)_ = 3.09, *P* = 0.08	2.58 (1.13 - 5.83)^*^	X^2^_(1)_ = 5.89, *P* = 0.015
Preoperative Plt.	0.97 (0.99 - 1.00)^*^	X^2^_(1)_ = 4.36, *P* = 0.04	1.00 (0.99 - 1.00)^*^	X^2^_(1)_ = 4.34, *P* = 0.037
eGFR	0.99 (0.98 - 1.01)^NS^	X^2^_(1)_ = 1.16, *P* = 0.28	Removed	Removed
BMI	0.86 (0.80 - 0.94)^***^	X^2^_(1)_ = 14.3, *P* < .001	0.86 (0.80 - 0.93)^***^	X^2^_(1)_ = 15.2, *P* < 0.001
Emergency surgery	0.77 (0.29 - 2.11)^NS^	X^2^_(1)_ = 0.25, *P* = 0.61	Removed	Removed

LR test statistics (Omnibus) and *P*-value:

* *P*-value (^NS^nonsense,

* *P* < 0.05,

*** *P* < 0.001) of Wald test statistics of logistic regression analysis

BMI=body mass index; CI=confidence interval; E-CABG=European Multicenter Study on Coronary Artery Bypass Grafting; eGFR=estimated glomerular filtration rate; Hb=hemoglobin; LR=likelihood ratio; OR=odds ratio; Plt.=platelet

**Table 5:** Risk scores in prediction of postoperative bleeding based on risk factors determined in multivariate analysis.

	Prediction of E-CABG grades 2 - 3			Prediction of drainage > 850 cc		
Risk groups	Predictors (scoring)	Crude incidence	Adj. HR (95% CI)	Predictors (scoring)	Crude incidence	Adj. HR (95% CI)
	Female (1)			Female (1)		
	BMI < 27 (1)			BMI < 27 (1)		
				Plt. count < 211 (1)		
Low risk	0 point	2.04% (1/49)		0 point	2.6% (1/39)	
Moderate risk	1 point	4.51% (6/133)	2.27 (0.26 - 19.33)	1 point	13.9% (12/86)	3.0 (0.64 - 14.11)
High risk	2 points	26.15% (30/115)	16.9 (2.24 - 128.1)	2 points	31.5% (39/124)	8.49 (1.95 - 37.01)
				3 points	54.2% (26/48)	21.86 (4.73 - 101.16)

Adj. HR=adjusted hazard ratio; BMI=body mass index; CI=confidence interval; E-CABG=European Multicenter Study on Coronary Artery Bypass Grafting; Plt.=platelet

In addition, the multivariate regression analysis revealed that female gender (adj. HR: 2.58; 95% CI: 1.13 - 5.83, *P* = 0.015), preoperative platelet values (adj. HR: 1.00; 95% CI: 0.99 - 1.00, *P* = 0.037), and BMI (adj. HR: 0.86; 95% CI: 0.80 - 0.93, *P* < 0.001) were significant predictors of bleeding according to the amount of drainage. After multivariate analysis, the cutoff value of 211×103 was used to categorize the platelet, and 27 was used for BMI according to ROC analysis. To have none of these predictors was considered as being in the low-risk group with a rate of 2.6% (1/39), to have only one of the predictors was considered a moderate risk (13.9, 12/86), and to have two (31.5%, 39/124) or more (54.2%, 26/48) of the predictors was considered a high risk regarding the prediction of drainage amount > 850 cc. Although there were more patients with excessive drainage than those in the low-risk group, being in the moderate-risk group does not increase the likelihood of excessive drainage (HR: 3.0, 95% CI: 0.64 - 14.11, *P* = 0.1642). As expected, having two or more of the predictors (high-risk group) increases the likelihood of excessive drainage significantly (HR: 8.49, 95% CI: 1.95 - 37.01, *P* = 0.0044, and HR: 21.86, 95% CI: 4.73 - 101.16, *P* < 0.0001, respectively)

Examinations of risk scores (Table 6) determined that the Papworth score was significant for E-CABG grades 2 - 3 group (*P* = 0.03). In our study, other scoring systems were not significant in predicting postoperative bleeding. However, all bleeding risk scores were insignificant in terms of drainage amount. Among the scoring parameters, preoperative hemogram (or hematocrit) value, platelet count, creatine (or eGFR), female gender, and antiplatelet use could be included into ORS.

**Table 6:** Univariate analysis of risk scores. Predictors of E-CABG 2 - 3 and drainage ≥ 850 ml/day.

Assessments of risk scores for E-CABG grades 2 - 3						
	Estimate	SE	HR	95% CI		*P*-value
				LE	UE	
Papworth	0.592	0.273	1.807	1.057	3.089	0.030
TRUST	0.055	0.139	1.057	0.805	1.388	0.689
TRACK	-0.023	0.032	0.977	0.918	1.041	0.476
WILL-BLEED	-0.050	0.057	0.952	0.851	1.064	0.385
ACTA-PORT	-0.013	0.041	0.987	0.911	1.070	0.758
Assessments of risk scores for drainage ≥ 850 ml						
	Estimate	SE	HR	95% CI		*P*-value
				LE	UE	
Papworth	0.365	0.218	1.441	0.940	2.209	0.094
TRUST	-0.074	0.105	0.928	0.755	1.141	0.481
TRACK	-0.182	0.023	0.999	0.955	1.045	0.965
WILL-BLEED	-0.050	0.042	0.951	0.876	1.034	0.238
ACTA-PORT	0.006	0.030	1.006	0.947	1.067	0.855

ACTA-PORT=Association of Cardiothoracic Anesthetists perioperative risk of blood transfusion; CI=confidence interval; E-CABG=European Multicenter Study on Coronary Artery Bypass Grafting; HR=hazard ratio; LE=lower end; SE=standard error; TRACK=Transfusion Risk and Clinical Knowledge; TRUST=Transfusion Risk Understanding Scoring Tool; UE=upper end

## DISCUSSION

After CABG operations became routine procedures in many centers, various scientific studies were conducted on each phase of CABG. We now know that postoperative bleeding is a serious cause of mortality and morbidity^[20-22]^. Bleeding may cause end-organ damage due to low perfusion, which may lead to increased ICU stay and hospital costs, cerebrovascular events, renal failure, mesentery ischemia, liver damage, and, ultimately, mortality^[20-22]^. Therefore, postoperative bleeding is one of the nightmares of surgeons. Blood product transfusion, performed to avoid these complications caused by hypovolemia and low oxygen supply, is a risky procedure itself. Studies have shown that febrile reactions, renal dysfunction, respiratory distress, immunosuppression, infections, and even low cardiac output can occur after blood product transfusion^[23,24]^. Our aim in this study was not to reprove all this information, but by including only diabetic patients treated with CABG, to predict more clearly the risk of bleeding in the early period in a limited patient population. The progression of massive bleeding is clear. The reason we included only diabetic patients in our study is that diabetes is one of the biggest vascular damage predictors^[25]^, its association with atherosclerotic heart disease is high^[26]^, and cardiovascular disease is the cause of mortality in approximately 75% of diabetic patients^[27]^.

The mean BMI values of patients in the E-CABG grades 2 - 3 group is lower than of those in grade 1 group - in Papworth risk scoring, low BMI is a risk factor for postoperative bleeding^[13]^. Frankly, we could not find the mechanism explaining the relationship between BMI and bleeding in our literature review. However, BMI is calculated as kg/m^2^, and a larger BMI indicates a larger body surface area. The amount of circulating blood per unit decreases due to the large body surface area. In addition, the ratio of the length of the sternotomy incision performed during CABG to the whole body may be a crucial factor in non-surgical bleeding, as it will correspond to a higher body ratio in patients with low BMI. Contrary to the previous finding, in the ≥ 850 ml/day group, BMI was directly proportionate with the amount of drainage. In other words, in E-CABG grades 2 - 3 group, low BMI is considered a factor for increased bleeding and transfusion requirement, but high BMI increases the risk for bleeding and transfusion requirement in the ≥ 850 ml/day drainage group. More studies on BMI and bleeding seem essential to resolve this confusion, by the results of which, BMI may be included in the ORS as a risk parameter.

The cornerstone of E-CABG is erythrocyte transfusion. In our study, both postoperative bleeding and erythrocyte, thrombocyte, and FFP transfusion rates were higher in grades 2 - 3 group. In the grouping made according to the amount of drainage, the use of all blood products in the drainage ≥ 850 ml/day group is high. This is expected because blood products are frequently used to stabilize hemodynamics in patients who are bleeding, while the risks of using blood products are often overlooked. These findings in our study are consistent with the literature^[1,3]^. It can be suggested that patients with normal preoperative hemogram (or hematocrit) values will need less transfusion in the postoperative period. The number of platelets, which are the basic elements of the coagulation system, and their functional capacity are also effective on postoperative bleeding^[28-30]^. In our study, platelet counts were significantly lower in the preoperative period in both the E-CABG grades 2 - 3 group and the ≥ 850 ml/day drainage group. In the light of these findings, we think that Hb (or hematocrit) and platelet values can be included in the ORS.

Our study included on-pump CABG patients. It is a known fact that the heart-lung machine causes end-organ damage^[31]^ due to changes in microcirculation and blood pressure, as well as microthrombi^[32,33]^. Accordingly, postoperative creatinine values were high in both E-CABG grades 2 - 3 and drainage ≥ 850 ml/day groups. Whether the increase in creatine values causes the tendency to bleed or creatine rises due to bleeding may be difficult to answer completely and accurately. Hypovolemia and hypotension due to bleeding may increase creatine values, but the tendency to bleed is 1.5 times higher in patients with renal failure in the preoperative period compared to patients with normal renal functions^[34]^, and its mechanism is still not fully elucidated. This reminds us once again of the effect of kidneys on hemodynamics. In our study, the preoperative creatinine clearance was significantly lower in the ≥ 850 ml/day drainage group. Therefore, we think that renal functions are associated with postoperative bleeding and deserve to be included in the ORS list. Among the bleeding scores, only WILL-BLEED and TRUST scores examine kidney functions. We believe this to be a deficiency of Papworth and TRACK scores.

There may be differences between genders in terms of clinical course and diseases. In the evaluation of postoperative bleeding, the female gender was at higher risk^[35,36]^. In our study, the female gender ratio was significantly higher in both E-CABG grades 2 - 3 group and ≥ 850 ml/day drainage group. Hence, it is our opinion that gender, which is not considered in Papworth, should be included in the ORS.

### Limitations

Our study has inherent limitations caused by its retrospective and single-center design. Age, hypertension, pulmonary hypertension, chronic pulmonary disease, and peripheral vascular diseases were not associated with postoperative bleeding. Interestingly, no significant results were obtained in terms of the tendency to bleed postoperatively in patients who were taken to emergency surgery. Antiplatelet agents are one of the main therapeutic agents in coronary artery diseases^[37]^. While discontinuation of acetylsalicylic acid (ASA) before the operation is not recommended, clopidogrel and the less frequently used ticagrelor should be discontinued at least five days before the operation^[37]^. Patients receiving ticagrelor were not included in the study. However, those using clopidogrel and ASA were not evaluated in separate groups. This can be considered the biggest limitation of our study. We can attribute the lack of statistically significant bleeding in patients undergoing emergency surgery to two reasons: the fact that the number of patients using clopidogrel and undergoing emergency operation is lower than those using ASA, and patients undergoing emergency surgery have recently been diagnosed with coronary artery disease and, therefore, have not received antiplatelet therapy before the operation. Ultimately, as clearly shown in the guidelines, the amount of postoperative bleeding may vary depending on the type of antiplatelet agent, and this is a proven fact that cannot be ignored for the timing of the operation^[37]^. Therefore, preoperative use of antiplatelets will be included in our ORS, which we plan to present with larger case numbers in the future.

Although we tried to be objective in this study, there may be deficiencies in data collection since it was designed retrospectively. We were able to get information about the additional diseases of the patients only as stated in the patient’s file. Therefore, it is not known whether the patients have hematological diseases such as hemophilia, factor 5 mutation, etc., and this may change the study results. The most accurate results can be achieved by prospectively re-running the study.

In addition, it is not known how effectively the thoracic cavity is aspirated before the operation is completed and the sternum is closed. Additionally, the amount of blood or coagula detected in surgical re-exploration was not added to the actual drainage volume. The use of other antiaggregants or anticoagulants such as ASA, clopidogrel, or new oral anticoagulants is unknown. Failure to do a thromboelastogram before administering FFP and RBC to patients can also be considered as a deficiency. All these are the negative aspects of retrospective studies and, unfortunately, they are also valid for our study too. This study constitutes the first step for the “optimal risk score” that we plan to use in predicting bleeding. We believe that the abovementioned limitations will be minimized in the prospective study that we plan to do as a second step.

## CONCLUSION

Current risk scores have been created for all open-heart surgery operations. Therefore, scoring may not yield accurate results for every type of surgery. We think that specific risk scores are needed for isolated CABG for a smoother surgical recovery process.

## Data Availability

The authors declare that the data supporting the findings of this study are available within the article and that data sharing is not applicable to this article.
